# A randomized feasibility pilot-study of intravenous and subcutaneous administration of ketamine to prevent postpartum depression after planned cesarean delivery under neuraxial anesthesia

**DOI:** 10.1186/s12884-022-05118-8

**Published:** 2022-10-21

**Authors:** David Thomas Monks, Arvind Palanisamy, Danish Jaffer, Preet Mohinder Singh, Ebony Carter, Shannon Lenze

**Affiliations:** 1 Department of Anesthesiology, Washington University School of Medicine, 660 S Euclid Avenue, St. Louis, MO 63110 USA; 2 Department of Obstetrics and Gynecology, Washington University School of Medicine, St. Louis, USA; 3 Department of Psychiatry, Washington University School of Medicine, St. Louis, USA

**Keywords:** Postpartum depression, Perinatal depression, Ketamine, Cesarean delivery

## Abstract

**Background:**

Evidence suggests ketamine may prevent postpartum depression (PPD) after cesarean delivery (CD) although intolerability and inconvenience of administration are problematic. We assessed the feasibility of studying ketamine (0.5 mg/kg, via subcutaneous injection or 40-min intravenous infusion) to prevent PPD after CD.

**Methods:**

Twenty-three women scheduled for cesarean delivery under neuraxial anesthesia were randomized to one of three groups: subcutaneous ketamine (SC Group, n = 8), intravenous ketamine (IV Group, *n* = 8) or placebo (*n* = 7). We measured depression (Edinburgh Postpartum Depression Scale [EPDS]) scores pre-operatively and at 1, 2, 21 and 42 days postoperatively. Anxiety, adverse effects, surgical site pain and analgesic consumption were also assessed. Feasibility was assessed based on acceptability, burden of disease, ability to collect study data and, tolerability of interventions.

**Results:**

Baseline characteristics of groups were similar, however, more women in the placebo group had pre-existing anxiety disorder (*p* = 0.03). 20.7% (25/121) of those approached consented to participate and 34.8% (8/23), of those assessed, screened positive for depression in the postpartum (EPDS > 12). PPD screening data was complete in 78.3% (18/23). No differences were observed for any adverse effect outcomes except for fewer incidences of intraoperative shivering with ketamine (SC: 25%, IV: 0% and Placebo: 85.7%, *p* = 0.01). No statistically significant difference in positive screening for PPD was observed (SC: 14.3%, IV: 50% and Placebo: 42.9%, *p* = 0.58).

**Conclusion:**

An RCT was judged to be feasible and there was no evidence of intolerability of either route of ketamine administration. Dispensing with the need for intravenous access makes the subcutaneous route a particularly attractive option for use in the postpartum population. Further examination of these interventions to prevent, and possibly treat, postpartum depression is warranted.

**Trial registration:**

NCT04227704, January 14^th^, 2020.

**Supplementary Information:**

The online version contains supplementary material available at 10.1186/s12884-022-05118-8.

## Background

Postpartum depression (PPD) complicates 6–19% of pregnancies, leading to costly consequences for mothers, their families, and the wider society [1]. One in eight affected women continue to be depressed at 2 years and there is a substantial financial burden to society with an estimated annual cost of $2.8 billion in the USA [2, 3].

Evidence is emerging regarding a potential role for ketamine in preventing PPD after cesarean delivery (CD). One study (*n*= 654) documented a reduction (12.8 vs. 19.6%) in the 6-week prevalence of PPD after a 48-h intravenous (IV) infusion (0.04 mg/kg/hr) of ketamine although three studies have shown no more than a marginal benefit with IV bolus administration of 0.25—0.5 mg/kg [4–7]. These trials also, however, demonstrated an increase in adverse effects, such as dizziness and hallucinations. An additional disadvantage of a prolonged IV infusion is the continued need for IV access. Commonly, IV therapies are discontinued within 24 h post-cesarean. This practice is thought to be essential to enhance postoperative recovery and aid the mother’s bonding with their babies. So, while ketamine may hold some promise in the prevention of PPD, questions remain regarding the optimal administration strategy.

Novel ketamine administration regimes outside of the postpartum setting offer the opportunity to bypass these limitations. For example, a brief (40 min) IV infusion of ketamine (0.5 mg/kg) has been tested as an antidepressant regimen [8]. In addition, the subcutaneous (SC) route has been examined as a method of administering ketamine to treat depression [9]. Neither of these routes, however, has been assessed for their antidepressant properties in a postpartum population. The objective of our study was to assess the feasibility of a randomized controlled trial (RCT) by examining patient participation rates, the efficacy of ketamine in preventing PPD, and the tolerability of a 40-min IV infusion or SC injection of 0.5 mg/kg of ketamine.

## Methods

The design, conduct and reporting of this feasibility pilot study was in accordance with the relevant extension to the CONSORT statement [10]. The study and all experimental protocols were approved by the Washington University Institutional Review Board (IRB ID: 201910191) and registered on ClinicalTrials.gov (NCT04227704, 14/01/2020). All methods were carried out in accordance with IRB guidelines and regulations. Written informed consent was obtained from all participants.

### Study design and setting

We conducted a parallel-arm, quadruple-blinded, feasibility pilot study with 1:1:1 randomization. The study was performed between December 2020 and August 2021 in a tertiary-referral maternity unit within an academic hospital in Saint Louis, Missouri, USA.

### Participants

Eligible women were English-speaking, aged 18—45 years old and scheduled for cesarean delivery under neuraxial anesthesia. Potentially eligible women were identified with reference to the Labor and Delivery OR schedule and contacted by phone and/or in-person on the day of surgery. Exclusion criteria were multiple pregnancy, severe comorbidities, and a history of psychosis or allergy to ketamine. Baseline data was collected from consenting participants regarding age, weight and height, gestational age, ethnicity, insurance status, psychiatric history, psychosocial stress (Antenatal Risk Questionnaire [ANRQ]), anxiety (General Anxiety Disorder-7 [GAD-7]) and depression (Edinburgh Postpartum Depression Scale [EPDS]).

### Interventions

All participants received spinal anesthesia with 1.6 – 1.8 ml of 0.75% hyperbaric bupivacaine, 15 mcg of fentanyl and 100 mcg of preservative free morphine. Participants in each arm of the study received a subcutaneous (SC) injection (approximately 0.5–1.0 ml) and a 40-min intravenous (IV) infusion (40 ml) of study injectates shortly after delivery of their baby. The injectates consisted of either 0.9% sodium chloride (saline) or 0.5 mg/kg of ketamine depending on their group allocation. One group received SC ketamine and IV saline (SC group); one group received SC saline and IV ketamine (IV group); and one group received SC saline and IV saline (Placebo group).

### Outcome measures

The primary outcome measures were related to feasibility and were: 1) the recruitment rate; 2) the 6-week period prevalence of postpartum depression (EPDS > 12) across the study cohort; 3) the ability of the investigative team to collect a complete dataset; and 4) the tolerability of the interventions. The *Edinburgh Postpartum Depression Scale* is a set of ten questions assessing mood in the previous seven days. It is intended as a screening tool to identify women requiring psychiatric assessment for depression. Each symptom is scored on a numeric rating scale of none (0) to severe (3). A positive depression screen was defined as EPDS > 12. The *General Anxiety Disorder-7* is a commonly used seven-item measure of general anxiety symptoms across various settings and populations. The *Antenatal Risk Questionnaire* is a patient-reported psychosocial assessment instrument aimed to predict those women who will go on to develop postpartum depression and which includes questions related to social support and history of emotional and sexual abuse.

Intraoperative data included delivery details, neonatal disposition, medications, maximum pain, vital signs, and adverse effects (nausea, vomiting, shivering, sedation, blurred vision, diplopia, dizziness, anxiety, pruritus, euphoria, amnesia, hallucinations, and nystagmus). Data was collected regarding anxiety (GAD-7), depression (EPDS), and surgical site pain on days 1, 2, 21 and 42. Additional postoperative data included successful establishment of breastfeeding, opiate consumption in the first 48 postoperative hours (oral morphine milligram equivalents), and adverse effects. Venous blood was collected from a dedicated cannula in the opposite arm to that used for the administration of the interventions, immediately before delivery of the baby and 20, 40 and 100 min after intervention administration.

All pain assessments were performed using a numerical rating score of 0—10, where a score of “0” was considered to indicate no pain whilst “10” was considered to indicate the worst pain imaginable. Inpatient assessments were performed either in-person or by telephone whilst out-patient assessments were exclusively performed by telephone.

### Statistical analysis

SPSS (version 25 for windows, IBM Inc., Chicago, USA) was used for statistical analysis. An alpha error of 5% was chosen as the threshold of statistical significance. Continuous data were analyzed using repeated measures ANOVA for time sequence variables. Student t test was used to compare pairwise data. Frequency data were compared using Chi square test with Yate’s correction when necessary. No formal sample size calculation was performed. An arbitrary a priori decision was made to enroll 60 participants. Participants were allocated to group chronologically according to a software-generated random sequence with permuted blocks of six. This was performed by the Investigational Drug Service who also prepared the study injectates but were otherwise uninvolved in the study. Participants, healthcare providers, outcome assessors and investigators were blinded to intervention allocation. Study injectates for each group allocation were identical in appearance.

## Results

**Fig. 1:**
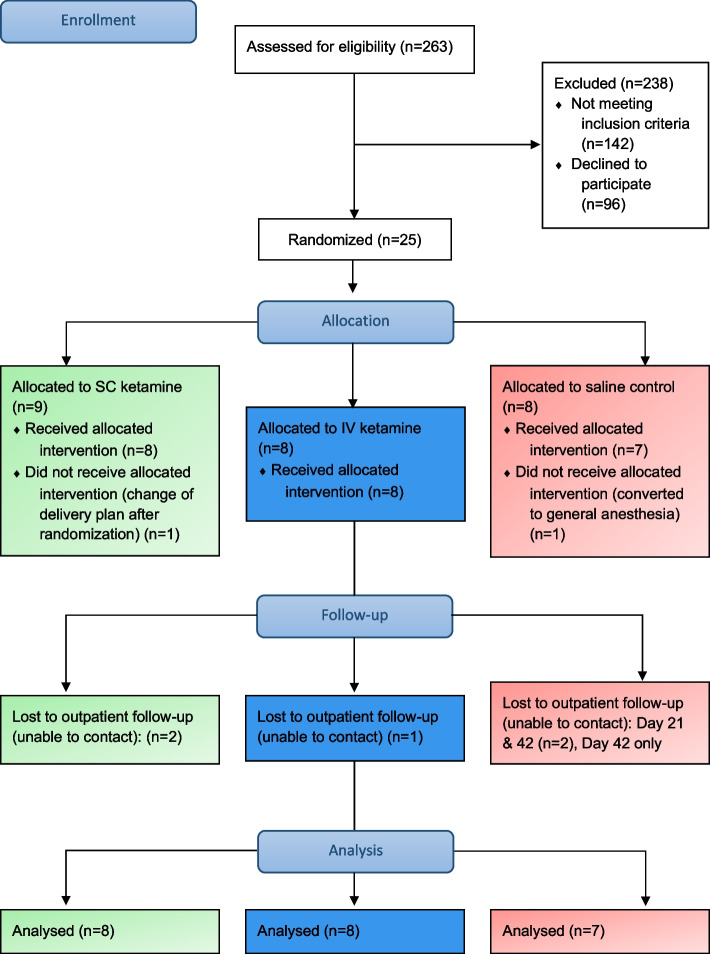
CONSORT Flow diagram

We approached 121 women to provide information about the trial and assess interest in participation. 25 women consented to participate in the study, of whom, two were withdrawn before receiving the intervention. These withdrawals were in one case due to failed neuraxial anesthesia, and in the other case the operation was cancelled in the operating room after successful manipulation of fetal position from breech to vertex. Eight women were allocated to each of the intervention groups while seven women were allocated to placebo control. Recruitment ceased, due to budgetary constraints, before meeting the arbitrarily chosen sample size. Also due to lack of funds at completion of the study, a planned analysis of plasma ketamine concentrations was not performed. The CONSORT flow diagram is shown in Fig. 1.

### Characteristics at baseline

There were no significant differences in baseline demographic characteristics between groups (Table 1). There were, however, more women in the placebo group with a pre-existing diagnosis of anxiety disorder (SC: 2/8 [25%]; IV: 2/8 [25%]; Placebo: 6/7 [85.7%], *p* = 0.03).

**Table 1:** Baseline characteristics

	**SC group (*n* = 8)**	**IV group (*n* = 8)**	**Placebo (*n* = 7)**	***P*-value**
**Age (years)**^a^	32.6 (0.95)	30.1 (4.30)	33 (6.53)	0.39
**Height (m)**^a^	1.64 (0.05)	1.62 (0.05)	1.63 (0.03)	0.65
**Weight (kg)**^a^	111.1 (34.9)	94.4 (12.4)	86.4 (19.0)	0.16
**BMI (kg/m**^**2**^**)**^a^	41.1 (12.0)	36.0 (6.06)	32.8 (7.84)	0.22
**Gestational age (weeks)**^a^	38.7 (1.11)	38.0 (1.11)	37.5 (0.76)	0.12
**American Society of Anesthesiologists (ASA) Physical Status (Classification I-V**^c^**, n (%))**^b^				0.68
ASA II	5 (62.5)	7 (87.5)	6 (85.7)	
ASA III	3 (37.5)	1 (12.5)	1 (14.3)	
**Ethnicity**^b^				0.86
Caucasian	5 (62.5)	6 (75)	5 (71.4)	
African American	3 (37.5)	2 (25)	1 (14.3)	
Hispanic	0 (0)	0 (0)	1 (14.3)	
**Insurance status**^b^				0.75
Insured	5 (62.5)	6 (75)	4 (57.1)	
Uninsured	3 (37.5)	2 (25)	3 (37.5)	
**Pre-operative screening**^a^				
Anxiety (GAD-7)	6.13 (8.20)	6.25 (5.55)	7.29 (2.93)	0.92
Depression (EPDS)	6.25 (6.27)	5 (4.78)	8.29 (4.72)	0.50
Psychosocial stress (ANRQ)	17 (14.4)	14.9 (9.25)	26.3 (13.0)	0.20
**Psychiatric history**^b^				
Any psychiatric history	2 (25)	1 (12.5)	7 (100)	0.05
PPD	2 (25.0)	0 (0)	2 (28.6)	0.78
MDD	1 (12.5)	0 (0)	3 (42.9)	0.36
Anxiety	2 (25.0)	2 (25.0)	6 (85.7)	0.03
Mood Disorder	0 (0)	0 (0)	2 (28.6)	-
Personality Disorder	0 (0)	0 (0)	0 (0)	-
Psychotic Disorder	0 (0)	0 (0)	0 (0)	-
Premenstrual Syndrome	0 (0)	0 (0)	0 (0)	-
Premenstrual Dysmorphic Disorder	0 (0)	0 (0)	0 (0)	-
Panic Disorder	0 (0)	1 (12.5)	0 (0)	-
Bipolar Affective Disorder	0 (0)	0 (0)	1 (14.3)	-
Obsessive Compulsive Disorder	0 (0)	0 (0)	1 (14.3)	-

Data are presented as: ^a^n (SD), ^b^n (%)

*GAD-7* General Anxiety Disorder scale, *EPDS* Edinburgh Postpartum Depression Scale, *ANRQ* Antenatal Risk Questionnaire

^c^American Society of Anesthesiologists (ASA) Physical Status Classification System is designed to assess and communicate a patient’s pre-anesthesia medical co-morbidities. A pregnant woman cannot be classified as ASA I, is classified as ASA II if they have no medical co-morbidities and is classified as ASA III if they have mild medical co-morbidities

### Feasibility measures

*Recruitment rate*: out of the 121 women who were approached, twenty-five (20.7%) consented to participation. *Burden of disease*: eight women (34.8%) screened positive for depressive symptoms on at least one postpartum assessment. *Data collection*: we obtained a full dataset for depression screening in 78.3% (18/23) in the 6-week study period and in all but one participant (95.7%) for inpatient assessments. *Tolerability*: adverse effects are shown in Table 2. No severe side effects were observed in any patients and the only difference was observed between groups was a greater frequency of intraoperative shivering in the placebo group compared with the ketamine intervention groups (*p* = 0.01).

**Table 2:** Adverse effects

	**SC group (*n* = 8)**	**IV Group (*n* = 8)**	**Placebo (*n* = 7)**	*P*-value*
**Self-reported AE incidence**				
	6 (75.0)	6 (75.0)	7 (100)	0.78
Nausea				
Mild	2 (25.0)	1 (12.5)	0 (0)	
Moderate	0 (0)	3 (37.5)	2 (28.6)	0.53
Severe	0 (0)	0 (0)	0 (0)	
Vomiting				
Mild	2 (25.0)	0 (0)	0 (0)	
Moderate	1 (12.5)	1 (12.5)	1 (14.3)	0.41
Severe	0 (0)	0 (0)	0 (0)	
Shivering				
Mild	1 (12.5)	0 (0)	6 (85.7)	
Moderate	1 (12.5)	0 (0)	0 (0)	0.01**
Severe	0 (0)	0 (0)	0 (0)	
Sedation				
Mild	1 (12.5)	2 (25.0)	1 (14.3)	
Moderate	0 (0)	1 (12.5)	2 (28.6)	0.38
Severe	0 (0)	0 (0)	0 (0)	
Blurred vision				
Mild	1 (12.5)	3 (37.5)	1 (14.3)	
Moderate	0 (0)	0 (0)	0 (0)	0.41
Severe	0 (0)	0 (0)	0 (0)	
Diplopia				
Mild	0 (0)	1 (12.5)	1 (14.3)	
Moderate	0 (0)	0 (0)	0 (0)	0.99**
Severe	0 (0)	0 (0)	0 (0)	
Dizziness				
Mild	1 (12.5)	3 (37.5)	1 (14.3)	
Moderate	0 (0)	0 (0)	0 (0)	0.41
Severe	0 (0)	0 (0)	0 (0)	
Anxiety				
Mild	0 (0)	1 (12.5)	2 (28.6)	
Moderate	0 (0)	0 (0)	1 (14.3)	0.23**
Severe	0 (0)	0 (0)	0 (0)	
Pruritus				
Mild	4 (50.0)	0 (0)	1 (14.3)	
Moderate	0 (0)	2 (25.0)	1 (14.3)	0.53
Severe	0 (0)	0 (0)	0 (0)	
Euphoria, Amnesia, Hallucinations, Nystagmus				
	0 (0)	0 (0)	0 (0)	
**Hemodynamic Adverse Effects**				
Hemodynamic AE incidence				
	3 (37.5)	3 (37.5)	2 (28.6)	0.92
Systolic Blood pressure				
< 80 mmHg	2 (25.0)	1 (12.5)	1 (14.3)	0.78
> 170 mmHg	0 (0)	1 (12.5)	1 (14.3)	0.68
Heart Rate				
< 40 bpm	0 (0)	2 (25.0)	0 (0)	0.70
> 120 bpm	1 (12.5)	1 (12.5)	0 (0)	0.99

Incidences are presented as n (%)

*SC* subcutaneous, *IV* intravenous, *AE* adverse effects

^*^Chi-squared test

^**^Chi-squared test, with Yates’ correction

### Postpartum mood, surgical site pain, opioid consumption, and breastfeeding

**Fig. 2:**
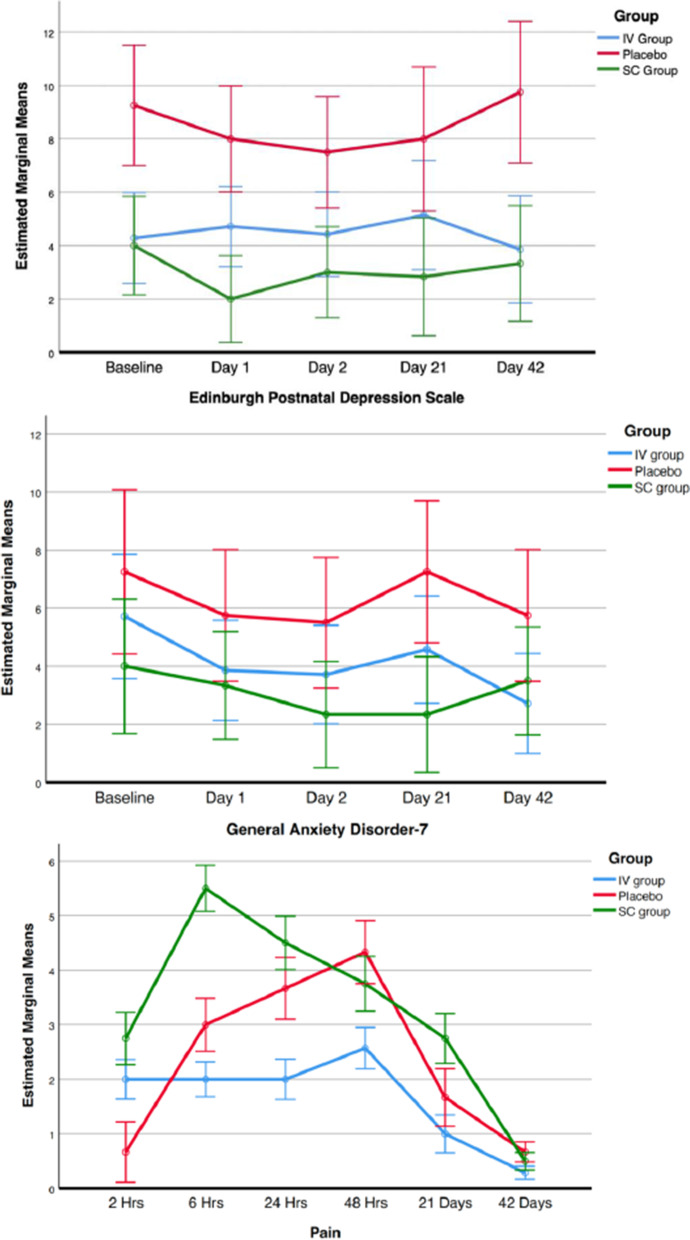
Graphs of depression, anxiety, and surgical site pain scores across the study period

The group mean EPDS, GAD-7 and pain scores at each timepoint from baseline to 42 days postpartum are shown in Fig. 2 (repeated measures ANOVA tests: EPDS, *p* = 0.08; GAD-7, *p* = 0.44; pain, *p* = 0.09). The mean morphine milligram equivalents consumed in the first 48 post-cesarean hours were 68.4 for the SC group, 67.0 for the IV group and 88.6 for the placebo (*p* = 0.83). No differences between groups were observed for breastfeeding success in the first 48 h post-cesarean (2/8 in SC group, 3/8 in IV group and 4/7 in placebo group, *p* = 0.56).

## Discussion

This pilot study demonstrates the feasibility of the use of ketamine, via subcutaneous injection or intravenous infusion, in our postpartum population and paves the way for an RCT powered to detect differences in the prevalence of postpartum depression.

Across all groups, almost 35% of women in our study screened positive for PPD (EPDS > 12). This is much higher than the prevalence of PPD observed in larger national cohorts. A positive screen does not necessarily result in a diagnosis of PPD upon formal psychiatric evaluation. Additionally, it is plausible that patients with higher levels of depression at presentation may find the intervention more attractive. Indeed, 17.4% (4/23) of women in our study had an EPDS > 12 at baseline.

Only 20.7% of eligible patients consented to participate. This low level of acceptability of the study will have undoubtedly been affected by some of the changes to the conduct of research that were necessary during the SARS-CoV-2 pandemic. It is also likely that some women may have felt that they had little to gain from potentially receiving another therapeutic agent with adverse effects. A further barrier to participation in this study is the need of an intravenous cannula. Administering ketamine subcutaneously has the advantage of not requiring IV access which could also make it a convenient intervention in postpartum women outside of the perioperative setting.

We found no evidence of intolerability of ketamine. There were no severe adverse effects experienced by patients receiving ketamine by either route. In fact, there was a reduction in shivering in the ketamine groups compared to the control, a finding that is in keeping with previous studies [11]. The convenience of the subcutaneous route for ketamine has led to its use as an antidepressant in non-pregnant patients and as a post-operative analgesic in a resource-poor setting [9, 12]. Despite this, it has not been examined for its antidepressant effects in the postpartum. We chose to examine these interventions, initially, in women, at low risk for depression, undergoing cesarean delivery with standard monitoring in the operating room. This facilitated the close assessment of participants symptoms and vital signs during administration. However, now that these interventions have shown themselves to be well tolerated, the way is paved for further examination of one or both interventions in a higher risk population outside of the operating room and possibly even in an outpatient setting.

Although we collected data on breastfeeding success, this study is potentially limited in its ability to assess neonatal safety outcomes. Encouragingly, early data suggests that intramuscular administration of doses up to 1.0 mg/kg of ketamine do not pass into the breast milk to a clinically significant extent (pre-print server, NCT04285684) which minimizes the possibility of adverse neonatal effects at the lower dose we used [13].

## Conclusions

In conclusion, an RCT of the anti-depressant effect of ketamine is feasible in our postpartum population and a dose of 0.5 mg/kg is well tolerated when administered as a 40-min infusion or subcutaneous injection. Dispensing with the need for intravenous access makes the subcutaneous route a particularly attractive option for use in the postpartum population. Further examination of these interventions to prevent postpartum depression is warranted.

## Supplementary Information

**Additional file 1.**

## Data Availability

The datasets supporting the conclusions of this article are included within the article (and its additional files).

## Abbreviations

PPD: Postpartum depression
CD: Cesarean delivery
SC: Subcutaneous
IV: Intravenous
GAD-7: General Anxiety Disorder scale
EPDS: Edinburgh Postpartum Depression Scale
ANRQ: Antenatal Risk Questionnaire
ASA: American Society of Anesthesiologists (Physical Status Classification system)
